# Cytoreduction (Peritonectomy Procedures) Combined with Hyperthermic Intraperitoneal Chemotherapy (HIPEC) in Advanced Ovarian Cancer: Retrospective Italian Multicenter Observational Study of 511 Cases

**DOI:** 10.1245/s10434-016-5686-1

**Published:** 2016-11-28

**Authors:** Angelo Di Giorgio, Pierandrea De Iaco, Michele De Simone, Alfredo Garofalo, Giovanni Scambia, Antonio Daniele Pinna, Giorgio Maria Verdecchia, Luca Ansaloni, Antonio Macrì, Paolo Cappellini, Valerio Ceriani, Giorgio Giorda, Daniele Biacchi, Marco Vaira, Mario Valle, Paolo Sammartino

**Affiliations:** 1 Department of Surgery ‘P. Valdoni’, Sapienza University of Rome, Rome, Italy; 2 General Surgery and Gynecologic Oncology Unit, Sant’Orsola Hospital, Bologna, Italy; 3 Unit of Surgical Oncology, Candiolo Cancer Institute, Turin, Italy; 4 Department of Surgery, Regina Elena National Cancer Institute, Rome, Italy; 5 Department of Obstetrics and Gynecology, Catholic University of the Sacred Heart, Rome, Italy; 6 Unit of Surgery and Advanced Oncologic Therapies, Morgagni-Pierantoni Hospital, Forlì, Italy; 7 General Surgery Unit, Papa Giovanni XXIII Hospital, Bergamo, Italy; 8 Department of Human Pathology, University of Messina, Messina, Italy; 9 General Surgery Unit, San Giovanni di Dio Hospital, Florence, Italy; 10 General Surgery UnitPoliclinico Polispecialistico Multimedica, Sesto San Giovanni, Italy; 11 Department of Gynecological Oncology, CRO National Cancer Institute, Aviano, Italy

## Abstract

**Purpose:**

The aim of this study was to help with the process of selecting patients with advanced ovarian cancer to undergo cytoreductive surgery (CRS) with hyperthermic intraperitoneal chemotherapy (HIPEC) by analyzing outcome data at distinct clinical time points reflecting the natural history of the disease.

**Methods:**

In a retrospective Italian multicenter study investigating patients with advanced ovarian cancer who underwent CRS plus HIPEC between 1998 and 2014, we analyzed data for consecutive patients at eight treatment time points: primary debulking surgery (PDS); interval debulking surgery after partial response, after no response, and after a pathologic complete response to neoadjuvant chemotherapy; first recurrence with a progression-free interval >12, <12 months, or >12 months in patients who underwent further chemotherapy before CRS and HIPEC; and patients who underwent two or more CRS procedures and chemotherapy lines before CRS and HIPEC.

**Results:**

The 511 enrolled patients underwent 3373 procedures; 72.6% achieved complete cytoreduction, with an overall major morbidity of 17.4%. At a median follow-up of 53.8 months, overall survival (OS) was 54.2 months (95% confidence interval [CI] 44–58.4) and progression-free (PFS) survival was 16.6 months (95% CI 14.7–19.1). Outcome analysis in patients in whom CRS plus HIPEC was used for primary advanced cancer or recurrent ovarian cancer showed significant differences in OS and PFS according to the time points analyzed. Multivariate analysis identified completeness of CRS, Peritoneal Cancer Index, and the times when patients underwent CRS plus HIPEC as independent prognostic factors.

**Conclusions:**

This selective information on survival should help in interpreting the findings from ongoing randomized studies focusing on CRS plus HIPEC in patients with advanced ovarian cancer.

Most patients with ovarian cancer are diagnosed at an advanced stage, and at least 75% of cases involve the peritoneum, frequently with ascites or subocclusion.^1^,^2^ Even though survival rates have improved over recent years,^2^–^5^ more than 70% of these patients have recurrent disease within 5 years.^6^–^8^

Given that ovarian disease remains confined within the peritoneal cavity for most of its clinical history, attention has turned towards aggressive locoregional therapy combining hyperthermic intraperitoneal chemotherapy (HIPEC) with maximal cytoreductive surgery (CRS) [peritonectomy procedures],^9^–^15^ an approach generally used in centers specifically involved in treating primary peritoneal tumors or peritoneal metastases from various origins, namely peritoneal surface malignancies (PSM).^16^–^18^ Despite numerous studies, including a randomized controlled trial and meta-analysis, showing better outcomes with acceptable morbidity after CRS combined with HIPEC than after traditional treatments for advanced ovarian cancer,^13^,^14^ wide skepticism persists about whether HIPEC really adds value to CRS alone, as well as concern that it might increase complications.^19^,^20^ A major hindrance to more widespread use of HIPEC combined with CRS in treating advanced ovarian cancer is that previous case series have mainly analyzed outcomes for two treatment settings (primary and recurrent disease), disregarding the long natural history of disease, multiple clinical scenarios and progressive disease stages.^21^,^22^ Hence, while we await the results from the numerous ongoing prospective randomized trials expected to provide data on the role of HIPEC combined with CRS in primary and recurrent disease,^23^ we now need to analyze outcome data at non-overlapping clinical time points related to patients’ responses to neoadjuvant chemotherapy (NACT) or adjuvant chemotherapy, and to the complex problems caused by repeated chemotherapy lines or CRS for multiple recurrence or disease progression. This valuable new information could help in the process of selecting patients to undergo CRS and HIPEC combined, and specify when the integrated procedure would have the greatest benefit on outcomes.

We designed this multicenter study to investigate a large series of patients with advanced ovarian cancer treated in the major Italian centers experienced in treating PSM with CRS and HIPEC combined. Our specific aim was to assess the results of the integrated procedure obtained in patients grouped according to primary and recurrent disease, and verify whether, within these settings, along with other prognostic variables, eight clinical time points reflecting surgical timing and responses to chemotherapy are independent prognostic factors. Outcome measures were morbidity, progression-free survival (PFS), and overall long-term survival during a median 5-year follow-up. Univariate and multivariate analyses were conducted to identify the most significant factors related to outcome.

## Methods

### Study Design

We conducted a retrospective, multicenter cohort study in 11 tertiary Italian centers experienced in treating PSM and ovarian cancers, over a 16-year period from December 1998 to December 2014. The Institutional Review Board for each center approved the study procedures.

### Patient Population

Data were collected by a single work group using a custom-designed database. We only collected data for patients whose records included complete information on clinical and epidemiological characteristics, including age, Eastern Cooperative Oncology Group (ECOG) performance status, tumor markers, diagnostic techniques, International Federation of Gynecology and Obstetrics (FIGO) stage,^24^ tumor histology,^25^ peritoneal disease spread according to the Peritoneal Cancer Index (PCI),^26^ surgical procedures used (including information on complications and operative mortality according to the Clavien–Dindo classification),^27^ CRS results according to the completeness of cytoreduction (CC) score,^26^ HIPEC techniques and drugs, number of adjuvant and NACT cycles, eventual drug-induced toxicity during systemic chemotherapy and HIPEC evaluated according to the National Cancer Institute Common Terminology Criteria for Adverse Events (CTCAE version 4.0)^28^ and last complete updated data on follow-up. Patients were grouped according to primary or recurrent disease when they underwent CRS and HIPEC. Each group was subdivided into four subgroups according to the various time points at which the disease was treated. Patients treated for primary disease (FIGO stage III tumors A, B, C and stage IVB) were subdivided as follows: Time 1, primary debulking surgery (PDS); Time 2, interval debulking surgery (IDS) after partial response to NACT; Time 3, IDS after no response to NACT (stable disease); and Time 4, IDS after a pathologic complete response (pCR) to NACT. NACT responses were evaluated according to the Response Evaluation Criteria in Solid Tumors (RECIST) revised guideline version 1.1,^29^ and pCR was assessed as proposed by Böhm et al.^30^ Patients treated for recurrent disease, regardless of FIGO stage at the primary operation, were subdivided as follows: Time 5, first recurrence with a progression-free interval >12 months; Time 6, first recurrence with a progression-free interval <12 months; Time 7, first recurrence with a progression-free interval >12 months in patients who underwent further chemotherapy before CRS and HIPEC; and Time 8, patients who underwent two or more CRS procedures for recurrence and two or more chemotherapy lines before CRS and HIPEC. Platinum-based chemotherapy sensitivity was defined according to the 2010 Gynecological Cancer Intergroup (GCIG) criteria.^31^ Indications for CRS plus HIPEC were peritoneal metastatic spread from advanced or recurrent ovarian cancer in patients younger than 75 years of age, with adequate cardiac, renal, hepatic and bone marrow function, ECOG performance status 0–2 with resectable disease, and written informed consent. Contraindications for CRS and HIPEC were extra-abdominal disease, other malignancies except breast cancer, unresectable disease, or patients who underwent NACT with progressive disease and patients whose severe associated medical conditions made them unfit for the procedure.

### Statistical Analysis

Follow-up data were completed on 31 December 2015. Patients with incomplete CRS (CC score >0) were considered as alive with disease at follow-up. Data were analyzed using the NCSS software package (2007; NCSS, LLC, Kaysville, UT, USA). The *χ* ^2^ test was used to analyze differences in frequencies and the *t* test was used to analyze differences among means. Multivariate logistic regression analysis was used to test risk factors for postoperative complications.

Overall survival (OS) was calculated from the date of CRS plus HIPEC to death or 31 December 2015, and PFS, with 95% confidence intervals (CI), was calculated to the date when disease recurred or progressed. Data for median follow-up were calculated as proposed by Schemper and Smith.^32^ Survival was analyzed using the Kaplan–Meier method and expressed as percentages to a maximum of 60 months, or as the median number of months. The log-rank test and Cox regression analysis were used for univariate and multivariate analysis of prognostic factors. In the univariate analyses, prognostic factors that correlated significantly with survival at least once were evaluated by multivariate Cox regression analysis. *p* values <0.05 were considered to indicate statistical significance.

## Results

Overall, 511 patients attending the 11 Italian centers met the inclusion criteria and underwent CRS and HIPEC—226 (44.2%) for primary advanced cancer and 285 (55.8%) for recurrent ovarian cancer (Table 1). All data supplied were reviewed by the senior surgeon (AD).

**Table 1:** Patient demographic and clinical characteristics listed according to the eight time points (511 patients)

Variables	All patients	Time 1	Time 2	Time 3	Time 4	Time 5	Time 6	Time 7	Time 8
	[*N* = 511, 100%]	[*n* = 53, 10.4%]	[*n* = 111, 21.7%]	[*n* = 45, 8.8%]	[*n* = 17, 3.3%]	[*n* = 95, 18.6%]	[*n* = 35, 6.9%]	[*n* = 49, 9.6%]	[*n* = 106, 20.7%]
Age [years; mean (range)]	57.1 (29–75)	60.4	60.1	58.3	60.7	53.7	54.8	57	54.8
BMI [mean (range)]	25.5 (14–44)	25	25	26.8	25.4	24.8	25.8	26.2	25.9
CA-125 **[**U/ml; mean (range)]	498.1 (1–12,000)	643	695.3	766	26.9	235.2	579	330	467
ECOG performance status									
0	219 (42.9)	21 (39.6)	46 (41.4)	18 (40)	13 (76.5)	49 (51.6)	19 (54.3)	17 (34.7)	36 (34)
1	203 (39.7)	21 (39.6)	50 (45.1)	16 (35.6)	3 (17.6)	37 (39)	13 (37.1)	20 (40.8)	43 (40.6)
2	89 (17.4)	11 (20.7)	15 (13.5)	11 (24.4)	1 (5.9)	9 (9.5)	3 (8.5)	12 (24.5)	27 (25.5)
Ascites	275 (53.8)	38 (71.7)	58 (52.2)	31 (68.9)	–	31 (32.6)	16 (45.7)	28 (57.1)	73 (68.9)
Histology									
Serous	443 (86.7)	46 (86.8)	101 (91)	39 (86.7)	17 (100)	81 (85.3)	29 (82.9)	35 (71.4)	95 (89.6)
Other	68 (13.3)	7 (13.2)	10 (9)	6 (12.4)	–	14 (14.7)	6 (17.1)	14 (28.6)	11 (10.4)
Grading									
High grade	419 (82)	40 (75.5)	85 (76.6)	20 (41.4)	17 (100)	85 (89.5)	32 (91.4)	44 (89.8)	81 (76.4)
Low grade	92 (18)	13 (24.5)	26 (23.4)	25 (55.6)	–	10 (10.5)	3 (8.5)	5 (10.2)	25 (23.6)

Data are expressed as *n* (%) unless otherwise stated

*BMI* body mass index, *ECOG* Eastern Cooperative Oncology Group

### Cytoreductive Surgery and Morbidity

At laparotomy, the mean PCI in the 511 patients was 12.7 (range 0–39), but differed significantly at the eight time points (*p* < 0.000 using the Student’s *t*-test). More surgical procedures were needed for patients with primary advanced cancer than for those with recurrent ovarian cancer (7.8 vs. 5.7). In 72.6% of patients, surgery achieved complete cytoreduction; the percentages for patients treated for primary advanced cancer (Times 1–4) and recurrent ovarian cancer (Times 5–8) were similar (70.8 vs. 74%). Surgery achieved the lowest percentage of complete cytoreduction in patients undergoing IDS and with no response to NACT. The data analysis comparing complete cytoreduction rates differed significantly at the eight time points (*p* < 0.013 using the *χ* ^2^ test) (Table 2). After surgery, patients were transferred to an intensive care unit (ICU) for a mean stay of 54 h (range 12–816). The mean hospital stay was 21 days (range 8–93), and the overall surgical morbidity rate was 44.2%. Of the 511 patients treated, 498 survived; overall operative mortality was 2.5% (13 cases). Multivariate logistic regression analysis identified a CC score > 0 and the need for more than four blood transfusions during surgery as significant risk factors for major complications (Table 3).

**Table 2:** Surgical procedures, and PCI and CC score listed according to the eight time points

	All patients [*n* = 511]	Time 1 [*n* = 53]	Time 2 [*n* = 111]	Time 3 [*n* = 45]	Time 4 [*n* = 17]	Time 5 [*n* = 95]	Time 6 [*n* = 35]	Time 7 [*n* = 49]	Time 8 [*n* = 106]	*p*-Value
*Surgical procedures*										
Peritonectomy procedures	1446	157	344	166	38	209	96	140	296	
Gynecological procedures (hysterectomy/adnexectomy/recurrent pelvic mass resection/vaginal resection)	257	53	111	45	17	4	8	7	12	
Gastrointestinal resections (gastric/small bowel/colorectal/appendix)	567	76	126	62	9	79	39	47	129	
Hepatobiliary and spleno-pancreatic resections	561	62	116	74	4	69	42	63	131	
Genitourinary resections (bladder/ureter)	35	3	5	5	–	8	2	2	10	
Lymphadenectomy (pelvic/para-aortic/inguinal)	507	69	145	46	21	61	21	45	99	
Total procedures	3373	420	847	398	89	430	208	304	677	
Mean procedures	6.6	7.9	7.6	8.8	5.2	4.5	5.9	6.2	6.4	
PCI [mean (range)]	12.7 (0–39)	15.8	11.6	16.1	5	10.2	13.9	13.8	14.4	**0.00002** ^a^
CC score										
CC 0 (%)	371 (72.6)	34 (64.2)	83 (74.8)	26 (57.8)	17 (100)	80 (84.2)	26 (74.3)	36 (73.5)	69 (65.1)	**0.013** ^b^
CC > 0 (%)	140 (27.4)	19 (35.8)	28 (25.2)	19 (42.2)	–	15 (15.8)	9 (25.7)	13 (26.5)	37 (34.9)	

*PCI* Peritoneal Cancer Index, *CC* completeness of cytoreduction

^a^ Using the T-test

^b^ Using the *χ* ^2^ test

**Table 3:** Anxnn

	All patients [*n* = 511]	Time 1 [*n* = 53]	Time 2 [*n* = 111]	Time 3 [*n* = 45]	Time 4 [*n* = 17]	Time 5 [*n* = 95]	Time 6 [*n* = 35]	Time 7 [*n* = 49]	Time 8 [*n* = 106]	*p*-Value
*Surgical morbidity (Clavien*–*Dindo classification) listed according to the eight time points*										
Morbidity										
Grade I–II (%)	137 (26.8)	13 (24.5)	41 (36.9)	13 (28.9)	3 (17.6)	18 (18.9)	11 (31.4)	9 (18.4)	29 (27.3)	NS
Grade ≥ III^a^ (%)	89 (17.4)	10 (18.9)	10 (9)	9 (20)	3 (17.6)	15 (15.8)	5 (14.3)	9 (18.4)	28 (26.4)	NS

	*p* value	OR	(Adjusted) 95% CI
*Risk factors for postoperative major morbidity (Grade III*–*IV), multivariate analyses (logistic regression)*			
Independent variables			
CC score 0 vs. > 0	**0.013**	0.24982	**0.29197–2.48208**
Duration of CRS + HIPEC (hours)			
≤8.6 vs. >8.6	0.047	0.79036	0.40447–0.87499
PCI ≤ 12.7 vs. > 12.7	0.854	0.93062	0.69562–0.83942
Blood transfusion units ≤4 vs. >4	**0.002**	0.35323	**0.37162–1.70963**

Bold values indicate statistical significance

*NS* non-significant, *CC* completeness of cytoreduction, *CRS* cytoreductive surgery, *HIPEC* hyperthermic intraperitoneal chemotherapy, *PCI* Peritoneal Cancer Index, *OR* odds ratio, *CI* confidence interval

^a^Including 13 cases (2.5%) of operative mortality

### Hyperthermic Intraperitonal Chemotherapy (HIPEC) and Systemic Post-HIPEC Chemotherapy

HIPEC was conducted using the closed technique in 53.8% of cases, the open technique in 23.9% of cases, and a semi-closed technique aided by a peritoneal cavity expander in 22.3% of cases. In 268 of the 511 patients (52.4%), HIPEC was administered with a single drug, i.e. cisplatin 75 mg/m^2^ for 60 min in 193 patients and oxaliplatin 460 mg/m^2^ for 30 min in 75 patients. In 243 patients (47.6%) cisplatin was combined with doxorubicin, paclitaxel, and mitomycin. HIPEC induced toxicity in 28 patients (5.4%): grade 1–2 acute kidney injury in 18 patients and grade 3 leukopenia in 10 patients, which was promptly reversed after medical treatment. Of the 498 patients who survived CRS plus HIPEC, 425 (85.3%) underwent systemic chemotherapy (patients who were considered platinum-sensitive received carboplatin and paclitaxel, and those who were considered platinum-resistant pegylated liposomal doxorubicin, topotecan, and, in recent years, biologic therapies) and 73 (14.6%) received no systemic chemotherapy for various reasons (unsuitable general conditions, toxicity, patient’s refusal).

### Histology

In most cases (86.7%), histological examination detected an ovarian papillary serous carcinoma in 82% high-grade cancers (Table 1). In the 332 patients who underwent lymphadenectomy, 41.6% had lymph node metastases.

### Survival

**Fig. 1:**
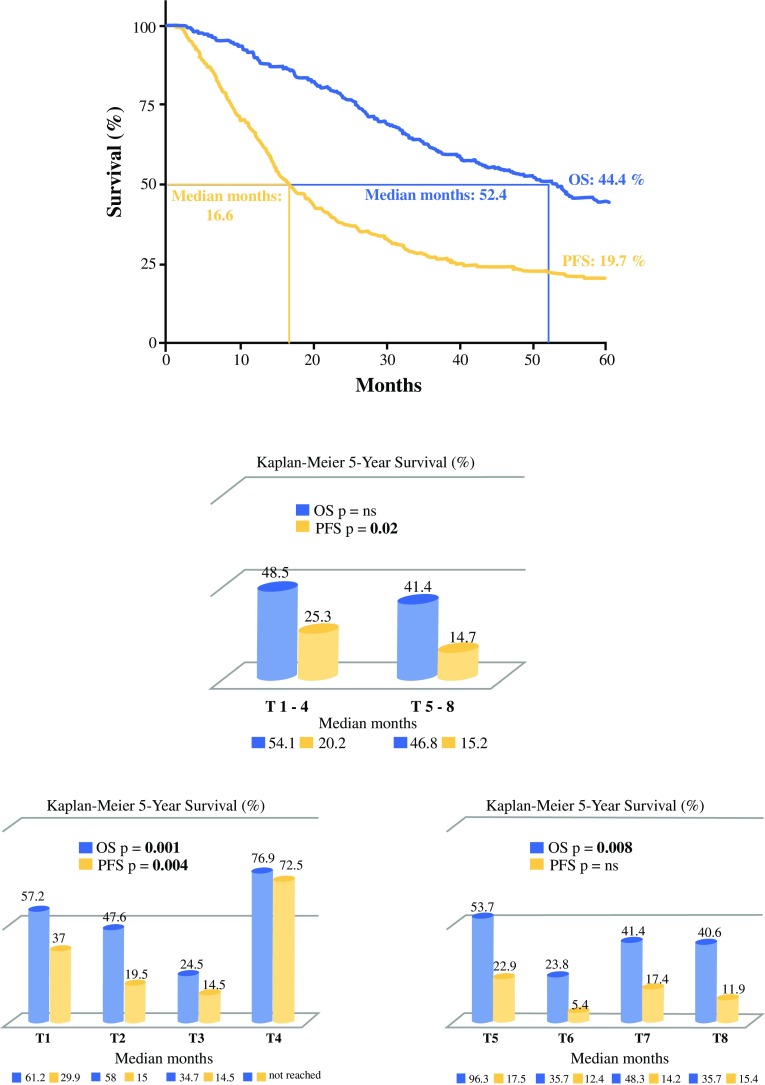
Overall survival and progression-free survival in the 511 patients treated with cytoreductive surgery and hyperthermic intraperitoneal chemotherapy. *OS* overall survival, *PFS* progression-free survival

At a mean follow-up of 53.8 months, 222 of the 511 patients enrolled in the study had died of disease (43.4%) [17 died of causes unrelated to advanced ovarian cancer (3.3%)] and 259 are still alive—130 (25.4%) with recurrent disease and 129 (25.2%) disease-free. Kaplan–Meier survival analysis indicated 5-year OS of 44.4% and PFS of 19.7%. Median OS was 52.4 months (95% CI 44.0–58.4) and PFS was 16.6 months (95% CI 14.7–19.1). Survival analysis showed a trend for better OS in patients treated for primary advanced ovarian cancer than in those treated for recurrence, but significantly better PFS. Outcome analysis in patients in whom CRS plus HIPEC was used for primary advanced ovarian cancer showed significant differences in OS and PFS according to the time points analyzed for that specific setting, especially in Time 3 (IDS after no response to NACT). Similarly, survival analysis in patients treated for recurrence showed that outcome differed significantly at the various time points when measured as OS rather than PFS, especially at Time 6 (first recurrence with a progression-free interval <12 months; Fig. 1). In the 511 patients, univariate analysis (log-rank test) identified CA-125 blood levels, ascites, extent of peritoneal spread (PCI), degree of cytoreduction achieved (CC score), tumor grading, and various time points when patients underwent CRS and HIPEC as variables significantly correlated with OS. Multivariate Cox regression analysis re-evaluating significant univariate prognostic factors, identified CC score, PCI and time points at which patients underwent CRS plus HIPEC as the most significant factors capable of independently influencing long-term survival (Table 4).

**Table 4:** Cox regression analysis of prognostic factors

Independent variables	Univariate	Multivariate		
	*p*-value	OR	(Adjusted) 95% CI	*p*-Value
CA-125 (≤498.1 vs. >498.1)	0.012	1.0481	0.7376–1.4892	0.793
Ascites (yes vs. no)	0.009	0.9409	0.6931–1.2773	0.696
PCI (≤12.7 vs. >12.7)	0.000	**1.9828**	**1.4435–2.7236**	**0.000**
CC score (0 vs. >0)	0.000	**1.6855**	**1.2305–2.3087**	**0.001**
Grading (high vs. low)	0.041	1.3361	0.9232–1.9336	0.124
Time (1–8)	0.000	**2.074**	**1.2637–3.4038**	**0.003**

Bold values indicate statistical significance

*OR* odds ratio, *CI* confidence interval, *PCI* Peritoneal Cancer Index, *CC* completeness of cytoreduction

## Discussion

In this Italian multicenter study conducted over 16 years in patients treated with CRS and HIPEC combined for advanced ovarian cancer, multivariate analysis showed that besides peritoneal spread (PCI) and CC score, another equally significant independent prognostic factor influencing outcome is the time when patients undergo CRS plus HIPEC. Even though our attempt to categorize CRS plus HIPEC-related outcomes according to biologic behaviors comes from retrospective data, our findings merit further research to refine the suggested profiles.

Possibly challenging other reports on the outcomes benefit of HIPEC,^10^,^22^,^33^ patients who underwent CRS plus HIPEC for primary advanced ovarian cancer had an almost similar or even better outcome than those treated for recurrence (Fig. 1). Our findings agree with published reports analyzing CRS with HIPEC,^13^,^15^ and compare well with those using primary CRS alone.^5^,^34^–^36^ Analyzing our outcomes and published data for patients in the two Gynecologic Oncology Group randomized control trials (114 and 172),^37^,^38^ our data, comparable mainly for patients at Time 1 (without NACT), compare well with those for both the control and normothermic intraperitoneal arms, especially given that our study sample mainly included patients with extensive peritoneal spread.

In patients treated for primary ovarian disease, in contrast to the results of the study by Chiva et al.,^34^ patients who underwent PDS (Time 1) and those who underwent IDS after NACT (Times 2–4), both combined with HIPEC, had a similar prognosis (OS 57.2 vs. 43.3%, median 61.2 vs. 53.2 months) [*p* = non-significant]. Extending current knowledge, patients who even partly responded to NACT (Time 2) had a significantly longer median number of months and OS than those who did not respond (Time 3; OS 47.6 vs. 24.5%, median 58 vs. 37.4 months; *p* < 0.007 using the *χ* ^2^ test; Fig. 1). In line with previous reports,^39^–^41^ the few patients in whom NACT achieved a pCR (Time 4) benefitted from particularly favorable survival after CRS and HIPEC, as already reported for PSM from colorectal cancer.^42^ Even though NACT is increasingly used as the primary treatment for advanced ovarian cancer,^35^ controversies persist on whether NACT might act as a driver for chemotherapy resistance.^43^,^44^ Our therapeutically useful finding that NACT responses significantly influence prognosis in patients who undergo CRS plus HIPEC leaves open to question whether and which patients with advanced ovarian cancer should undergo NACT (whenever not required by tumor burden). Nor does it specify whether NACT non-responders (Time 3) might benefit from HIPEC eventually combined with CRS.

For patients treated for recurrent disease, our collective results in patients with a high peritoneal disease burden compare well with the most recently published studies addressing secondary CRS without HIPEC, and also because many refer to localized ovarian recurrent disease.^45^–^48^ Pooled data for first platinum-sensitive recurrence (Times 5 and 7) show that, together, these patients have a significantly better OS than those treated for their first platinum-resistance recurrence (Time 6; 49.2 vs. 23.8%; *p* < 0.002 using the *χ* ^2^ test). Because our findings surprisingly argue against the reported benefits of CRS combined with HIPEC in platinum-resistant patients (Time 6),^10^,^14^ this question remains open to further research. Presumably, the long, 12-month cut-off we used to define platinum-based chemotherapy sensitivity allowed us to select truly chemotherapy-sensitive or resistant patients. Another useful finding came from our decision to analyze data for patients treated for first platinum-sensitive recurrence (Times 5 and 7) separately according to whether they had undergone further chemotherapy cycles before CRS and HIPEC (Fig. 1). Supporting previous findings,^49^ our outcome data therefore suggest that patients who have resectable platinum-sensitive recurrence should undergo surgery without further chemotherapy.

Last, by analyzing our data in patients with advanced ovarian cancer according to the long natural history of disease, another finding relates to the satisfactory OS (40.6%, median 35.7 months), our multicenter study reports in patients who underwent CRS and HIPEC after two or more CRS procedures and two or more chemotherapy lines for recurrence (Time 8). Our outcome findings compare well with published reports in patients who underwent tertiary and quaternary CRS without HIPEC, given that most patients had minimally extensive peritoneal disease.^50^–^52^

Apart from its retrospective design, a limitation of this study is that each cohort included few patients.

## Conclusions

The selective information on survival provided by this Italian multicenter study, assessed according to distinct time points in the natural history of disease, should simplify the process of selecting patients with advanced ovarian cancer to undergo HIPEC combined with CRS, specifying when this integrated procedure might have the greatest outcome benefit. Our results should help interpret findings from ongoing randomized studies investigating the two main settings—primary and recurrent disease—and may also suggest which patients to select to avoid bias in future randomized trials.
